# Molecular Engineering Strategies Tailoring the Apoptotic Response to a MET Therapeutic Antibody

**DOI:** 10.3390/cancers12030741

**Published:** 2020-03-21

**Authors:** Chiara Modica, Simona Gallo, Cristina Chiriaco, Martina Spilinga, Paolo Maria Comoglio, Tiziana Crepaldi, Cristina Basilico, Elisa Vigna

**Affiliations:** 1 Candiolo Cancer Institute, FPO-IRCCS, Strada Provinciale 142, 10060 Candiolo (TO), Italy; chiara.modica@ircc.it (C.M.); simona.gallo@ircc.it (S.G.); cristina.chiriaco@ircc.it (C.C.); martina.spilinga@ircc.it (M.S.); pcomoglio@gmail.com (P.M.C.); tiziana.crepaldi@ircc.it (T.C.); elisa.vigna@ircc.it (E.V.); 2 Department of Oncology, University of Turin, 10060 Candiolo, Italy

**Keywords:** *MET* oncogene, antibodies, apoptosis, MET targeted therapy

## Abstract

The *MET* oncogene encodes a tyrosine kinase receptor involved in the control of a complex network of biological responses that include protection from apoptosis and stimulation of cell growth during embryogenesis, tissue regeneration, and cancer progression. We previously developed an antagonist antibody (DN30) inducing the physical removal of the receptor from the cell surface and resulting in suppression of the biological responses to MET. In its bivalent form, the antibody displayed a residual agonist activity, due to dimerization of the lingering receptors, and partial activation of the downstream signaling cascade. The balance between the two opposing activities is variable in different biological systems and is hardly predictable. In this study, we generated and characterized two single-chain antibody fragments derived from DN30, sharing the same variable regions but including linkers different in length and composition. The two engineered molecules bind MET with high affinity but induce different biological responses. One behaves as a MET-antagonist, promoting programmed cell death in *MET* “addicted” cancer cells. The other acts as a hepatocyte growth factor (HGF)-mimetic, protecting normal cells from doxorubicin-induced apoptosis. Thus, by engineering the same receptor antibody, it is possible to generate molecules enhancing or inhibiting apoptosis either to kill cancer cells or to protect healthy tissues from the injuries of chemotherapy.

## 1. Introduction

The *MET* oncogene encodes a tyrosine kinase (hepatocyte growth factor (HGF) receptor) endowed with physiological roles during embryo development and organ regeneration. This role includes upregulation of cell motility, growth control, and protection from apoptosis [1]. In pathological conditions, *MET* is a dreadful driver oncogene for cancer onset and progression [2,3]. Aberrant activation of MET is often detected in human cancers, and may result from different molecular mechanisms, such as genetic alterations, receptor overexpression, and autocrine stimulation. A variety of genetic alterations of *MET* has been described, including: (i) gene amplification, that leads to receptor overexpression and constitutive activation, and is a feature of *MET*-addicted tumors; (ii) point mutations, that may cause ligand-independent kinase activation, anomalous signaling, resistance to target inhibitors, or may concur to the production of dysfunctional splicing variants; (iii) gene rearrangements, that generate aberrantly active fusion proteins such TPR-MET and PTPRZ1-MET [4]. The biological responses triggered by MET activation rely not only on its role as a modulator of cellular proliferation, but also on its involvement in the regulation of apoptotic process [5]. Apoptosis is a tightly regulated mechanism of cell death directed by a complex network of molecules belonging to the B-cell lymphoma 2 (BCL-2) protein family [6]. In cancer, inhibition of apoptosis is instrumental to shift the balance between cell death and proliferation in favor of the latter, thus implementing expansion of the neoplastic clone. MET activation provides strong anti-apoptotic stimuli through the phosphoinositide 3-kinase (PI3K)/AKT signaling pathway [7], by switching off the pro-apoptotic protein BCL-2 associated antagonist of cell death (BAD) and activating the E3 ubiquitin-protein ligaseMDM2, which promotes degradation of the pro-apoptotic protein p53 [8]. MET-dependent anti-apoptotic response is also achieved by activation of the mitogen-activated protein kinase (MAPK) signaling cascade, which turns on GATA binding protein 4 (GATA-4), a transcriptional factor upregulating the expression of the anti-apoptotic B-cell lymphoma-extra large (BCL-xL) protein [9].

In the past, we generated and characterized a MET antibody (DN30 mAb) with substantial antagonist activity, mostly due to the uncommon property of inducing release of MET from the cell surface through a proteolytic mechanism known as “shedding” [10]. However, the biological response to the native antibody was paradoxically “dual”, as it displayed a partial agonist activity in its bivalent form. The latter is due to dimerization and trans-phosphorylation of the few MET molecules left at the cell surface. Nevertheless, this residual receptor phosphorylation does not trigger the entire spectrum of MET downstream signaling. While the PI3K/AKT pathway is fully activated, a reduced and short-lasting extracellular signal-regulated kinase-1/2 (ERK-1/2) stimulation is induced [11]. As a result, only a subset of the pleiotropic MET-mediated biological responses is triggered; among those, protection from apoptosis. The balance between the two opposite activities elicited by the antibody varies in different biological systems and is hardly predictable. This dichotomy represents a limit for the therapeutic application of the native DN30 mAb, either as a MET inhibitor or as an HGF mimetic. The antagonist activity can be exploited in the treatment of some cancers (namely *MET*-addicted tumors [4]), while the agonist activity can find a therapeutic opportunity to mitigate apoptosis-mediated adverse effects of chemotherapy on healthy tissues [12].

Here we describe the generation and characterization of two single-chain Fabs, derived from DN30 mAb, differing from each other only by composition and length of the linkers connecting the light and heavy immunoglobulin chains. Both linkers have been previously used for the generation of recombinant antibody fragments or fusion proteins [13,14]. They share a flexible structure but are characterized by the presence of a different number and arrangement of hydrophilic or aliphatic amino acids that may influence the tridimensional conformation of the molecule. Through this engineering strategy, we built molecules with antagonist or agonist properties. These new molecules have potential therapeutic properties to control tumor growth or to protect healthy tissues from chemotherapy injuries, respectively.

## 2. Results

### 2.1. Generation of Single-Chain Fabs

**Figure 1:**
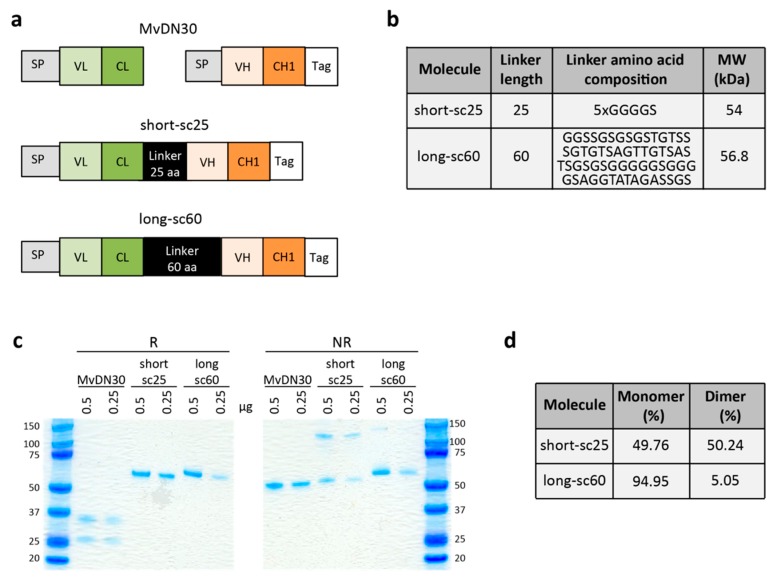
Schematic representation and analysis of single-chain Fabs. (**a**) Schematic representation of the parental MvDN30 and derived antibodies in single-chain format (short-sc25 and long-sc60). SP: signal peptide; VL: variable light domain derived from DN30; CL: constant light domain derived from human IgK light chain; VH: variable heavy domain derived from DN30; CH1: first constant heavy domain derived from human IgG1 heavy chain; Tag: strep and his tag sequences. (**b**) Table showing the amino acid sequences of the linkers included in the single-chain Fabs. The molecular weight (MW) of the two different single-chain antibodies calculated on the basis of their amino acid sequences (including the linker) is reported. (**c**) SDS-PAGE of the purified DN30 derivatives in reducing (R) and non-reducing (NR) conditions. (**d**) Table showing the percentage of the monomer and dimer forms of each single-chain antibody calculated by quantification of the SDS-PAGE bands.

Starting from the sequence of the chimeric Fab derived from DN30 (MvDN30) [15], we engineered two single-chain Fabs in which the C-terminus of the constant light (CL) domain and the N-terminus of the variable heavy (VH) domain are connected by an in-frame amino acid string. The resulting single polypeptide displays the following domain’s composition: VL-CL-linker-VH-CH1 (variable light-constant light-linker-variable heavy-first constant heavy). At the C-terminus of the sequence, a strep-tag and a poly-histidine tail were added for purification and detection purposes (Figure 1a). The two single-chain molecules differ from each other only by length and composition of the amino acid linker (Figure 1b): one is composed by a five-fold repeat of a GGGGS stretch, for a total of 25 amino acids (short-sc25), the other is a 60 amino acid-long sequence rich in G and S residues (long-sc60). By SDS-PAGE analysis under reducing conditions, a single band of the expected molecular weight (about 54 kDa for the short-sc25 and 57 kDa for the long-sc60) was detected, suggesting that the engineered molecules are indeed in single-chain form (Figure 1c). In non-reducing conditions, the single-chain Fab with shorter linker (short-sc25) displayed an additional band of about 120 kDa, accounting for around 50% of total protein, likely representing a dimer (Figure 1c). On the contrary, the single-chain Fab with the longer linker (long-sc60) showed a major band of 57 kDa accounting for about 95% of the total protein, suggesting that it maintains a monomeric format also in non-reducing conditions (Figure 1d).

### 2.2. Single-Chain Fabs Bind MET with High Affinity and Induce Shedding, but Differentially Modulate MET Phosphorylation

**Figure 2:**
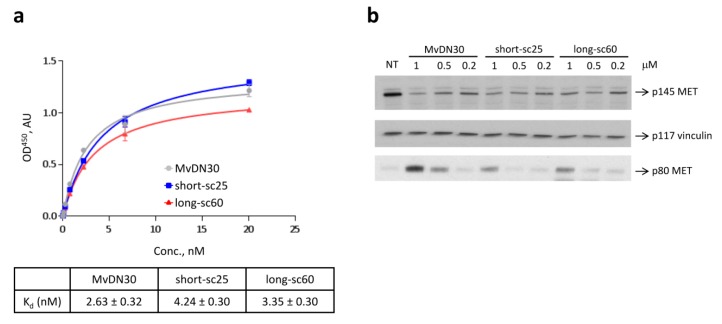
Single-chain Fabs maintain the ability to bind MET with high affinity and induce receptor shedding. (**a**) ELISA binding analysis of the interaction between MET and the single-chain Fabs. The extracellular region of MET fused to the Fc portion of a human IgG (MET-Fc chimera) was immobilized in solid phase and exposed to increasing concentrations of single-chain Fabs in liquid phase. MvDN30 was included in the assay as reference. Each point is the mean of triplicate values; bars represent standard deviation. OD^450^: optical density at 450 nm. AU: arbitrary units. (**b**) Immunoblotting analysis of cell lysates (top panels) and cell culture supernatants (bottom panel) from A549 cells treated for 48 h with the indicated concentrations of the single-chain Fabs; MvDN30 was included in the assay as reference. MET receptor and MET ectodomain levels were detected using anti-MET antibodies that bind epitopes located in the C-terminal tail or in the extracellular domain of the molecule, respectively. Vinculin (p117) was used as loading control. p145 MET: MET receptor β chain; p80 MET: MET ectodomain; NT: non-treated cells. Data reported in the figure are representative of at least two independent experiments.

To evaluate the binding of the two recombinant molecules to MET receptor, we performed an ELISA assay using the extracellular region of MET fused to the Fc portion of a human IgG (MET-Fc chimera) in solid phase, and in increasing concentrations of the single-chain Fabs in liquid phase. This analysis revealed that both short-sc25 and long-sc60 bind MET with an affinity comparable to the parental MvDN30 (Figure 2a). Similarly, the two recombinant antibodies induced MET shedding, although with reduced potency as compared to the parental MvDN30 (Figure 2b).

**Figure 3:**
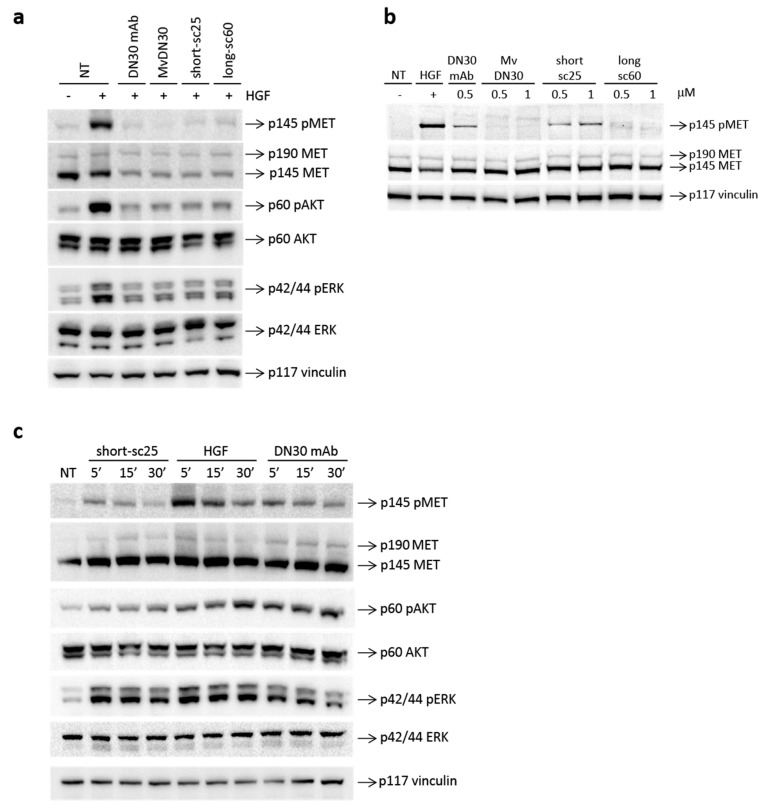
Single-chain Fabs inhibit HGF-dependent MET phosphorylation and differentially modulate MET activation. (**a**) Immunoblotting analysis of lysates from A549 cells treated for 24 h with single-chain Fabs (1 µM) and then stimulated with HGF (100 ng/mL) for 15 min; DN30 mAb and MvDN30 (1 µM) were included in the assay as reference. (**b**) Immunoblotting analysis of lysates from A549 cells treated for 15 min with the indicated concentrations of the single-chain Fabs; HGF, DN30 mAb, and MvDN30 were included in the assay as reference. (**c**) Immunoblotting analysis of lysates from A549 cells treated for different times with short-sc25 (0.5 μM), HGF (100 ng/mL), or DN30 mAb (0.5 μM). MET phosphorylation and total MET levels were measured using anti-MET antibodies recognizing the major phosphorylation site (Tyr^1234-1235^) or the C-terminal tail of the molecule, respectively. AKT phosphorylation was measured using anti-AKT antibodies recognizing the phosphorylated Ser473, and ERK phosphorylation was measured using anti-ERK antibodies recognizing the phosphorylated Thr202/Tyr204. Vinculin (p117) was used as loading control. p145 pMET: phosphorylated MET receptor β chain; p190 MET: unprocessed MET receptor; p145 MET: MET receptor β chain; p60 pAKT: phosphorylated AKT; p60 AKT: AKT; p42/42 pERK: phosphorylated ERK; p42/42 ERK: ERK; p117 vinculin: vinculin; NT: non-treated cells. Data reported in the figure are representative of at least three independent experiments.

The effect of single-chain Fabs on MET activation was analyzed in A549 cells, which express physiological levels of MET, and represent a standard to determine modulation of MET phosphorylation by exogenous factors [16]. To assess the antagonist activity, A549 cells were stimulated with HGF in the presence of the single-chain Fabs. The parental DN30 mAb and MvDN30 were used as the control. As shown in Figure 3a, all molecules impaired HGF-induced MET phosphorylation with similar potency. Accordingly, main MET downstream signal transducers AKT and ERK were also inhibited. To test possible ligand-mimetic activity, the experiment was performed in the absence of HGF. MvDN30, DN30 mAb, and HGF were included in the assay as controls. The results obtained in this assay indicate that the two single-chain antibodies behaved differently: while long-sc60 was a pure antagonist, short-sc25 induced a dose-dependent MET phosphorylation (Figure 3b). Quantification of the ratio between phosphorylated and total MET receptors is shown in Figure S1. To further explore the agonist properties of short-sc25, a time-course experiment was performed (Figure 3c). The natural ligand HGF strongly activated MET, displaying maximal activity at 5 min; DN30 mAb stimulated MET phosphorylation with less potency, and short-sc25 was the weakest agonist. This translated in a different activation of signal transducers. In particular, AKT phosphorylation was reduced, and ERK signaling was less sustained. Finally, we analyzed the activity of the short-sc25 in the presence of HGF. The addition of the antibody did not produce major modification of HGF-induced MET phosphorylation. The analysis of downstream signaling showed that the combination inhibited AKT activation, while ERK phosphorylation remained unaffected (Figure S2).

### 2.3. Single-Chain Fabs Elicit Different Effect on Cell Growth and Scattering

**Figure 4:**
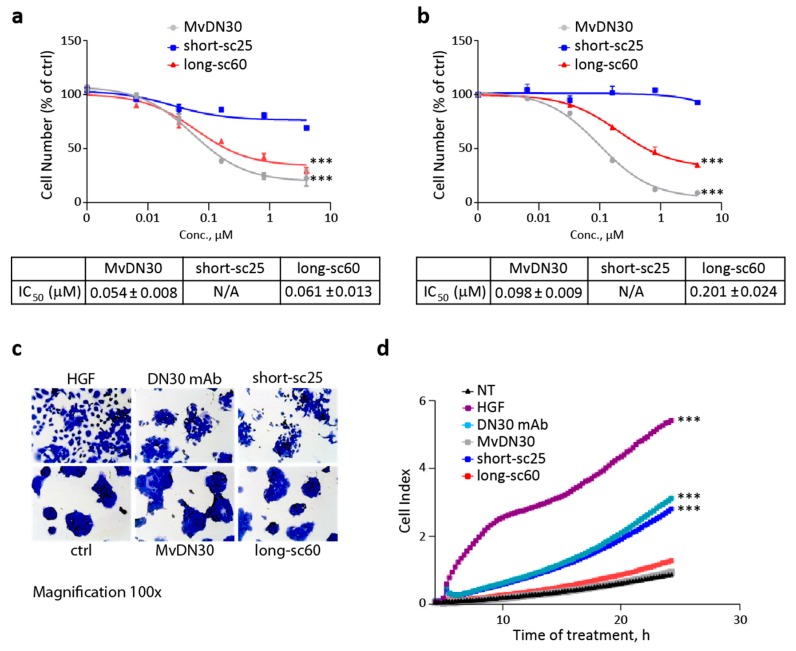
Long-sc60 inhibits *MET*-addicted cancer cell growth while short-sc25 induces cancer cell scattering. Growth of *MET*-addicted (**a**) SNU-5 and (**b**) EBC-1 cancer cells treated with increasing concentrations of the single-chain Fabs (0–5 µM) for 72 h. MvDN30 was included in the assay as reference. Graphs represent the percentage of living cells with respect to untreated cells. Samples are in triplicate; bars represent standard deviations. Scattering of human pancreatic adenocarcinoma (HPAF-II) cells in response to treatment with single-chain Fabs (100 nM) monitored (**c**) at end-point or (**d**) in real time using the X-CELLigence Real-Time Cell Analyzer (RTCA) device and expressed as normalized cell index. HGF (5 ng/mL), DN30 mAb (100 nM), and MvDN30 (100 nM) were included in the assay as references. NT: non-treated cells. Pictures and data reported in the plots are representative of at least three independent experiments. *** *p* < 0.001 t-test of treated versus untreated cells at the end of the experiments.

We next studied if the antagonist and agonist properties of the long-sc60 and short-sc25, respectively, translated in different biological responses. To test the activity of the recombinant molecules on the growth of MET-addicted cancer cells (i.e., relying on MET signaling for their growth) SNU-5 and EBC-1 cells [17] were incubated with increasing concentrations of the two single-chains, and cell growth was assessed after three days of culture. As shown in Figure 4a,b, long-sc60 strongly impaired the viability of both cancer cell lines, while short-sc25 was ineffective. The effect of the two single-chain derivatives was then analyzed by “scatter” assay, a prototype assay for MET activity [18]. Human pancreatic adenocarcinoma (HPAF-II) cells were treated for 24 h with the test compounds, and cell scattering was either evaluated at the end-point by microscopic analysis (Figure 4c), or followed in real time by measurement of the variation in electrical impedance of cell-covered electrodes (X-CELLigence Real-Time Cell Analyzer) (Figure 4d). The agonist short-sc25 induced HPAF-II cell scattering with a potency similar to the bivalent antibody DN30 mAb, while the long-sc60 did not induce any motility response, behaving as the native MvDN30. The HGF-mimetic activity of short-sc25 was confirmed in a scatter assay using A549 cells. As shown in Figure S3, short-sc25 induced cell scattering. The activity of the antibody was also tested in the presence of HGF; in this condition, short-sc25 inhibited ligand-induced cell motility.

### 2.4. Long-sc60 Induces Apoptosis in MET-Addicted Cancer Cells

**Figure 5:**
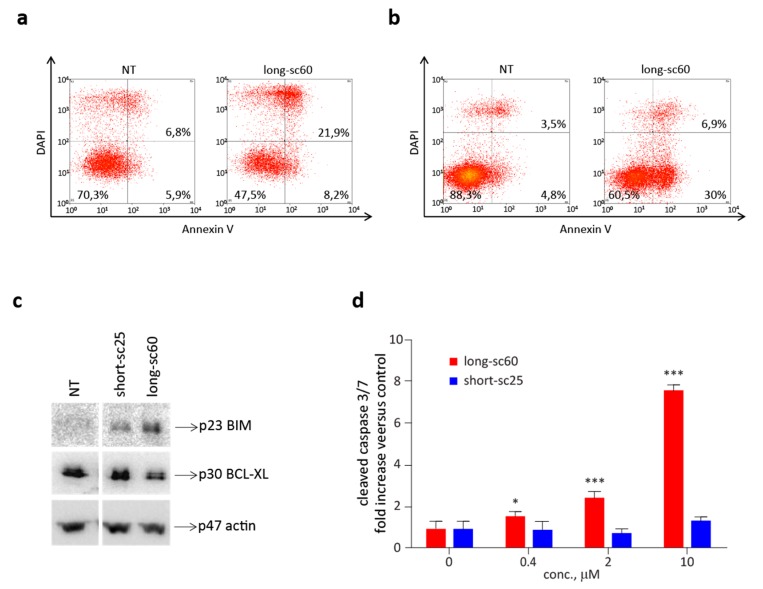
Long-sc60 induces apoptosis in MET-addicted cancer cells. Flow cytometry analysis of MET-addicted (**a**) SNU-5 or (**b**) EBC-1 cells treated with long-sc60 (0.5 µM) for 48 h and then stained with Annexin V-APC and DAPI. (**c**) Immunoblotting analysis of lysates from EBC-1 cells treated with long-sc60 or short-sc25 (0.5 µM); modulation of pro-apoptotic and anti-apoptotic pathways was determined evaluating the expression levels of BIM and BCL-xL, respectively. Actin (p47) was used as loading control. (**d**) Analysis of caspase-3 activation in EBC-1 cells treated with increasing concentrations of long-sc60 or short-sc25 for 72 h. Graph represents cleaved caspase-3 activity expressed as fold increase versus control. Samples are in triplicate; bars represent standard deviations. Data reported in the figure are representative of at least three independent experiments. *** *p* < 0.001; * *p* < 0.05 t-test of treated versus untreated cells at the end of the experiments.

Next, we focused on the ability of the long-sc60 to modulate cell apoptosis. As expected from its MET inhibitory properties, long-sc60 significantly blocked MET-mediated protection from apoptosis, as assessed by Annexin V assays performed in MET-addicted SNU-5 and EBC-1 cells (Figure 5a,b). The analysis of a panel of molecules involved in the regulation of apoptosis showed that long-sc60 activated pro-apoptotic signaling and concomitantly attenuated anti-apoptotic pathways. On one hand it upregulated the expression of the pro-apoptotic molecule BIM, and on the other hand it reduced the expression of BCL-xL, one of the main players in the negative regulation of programmed cell death (Figure 5c) [19,20]. On the contrary, short-sc25 did not influence the expression of BCL-xL, and only marginally induced BIM. Inhibition of MET-mediated anti-apoptotic activity was further confirmed by dose-dependent increment of cleaved caspase-3 (Figure 5d). The short-sc25 had no biological activity in this assay.

### 2.5. Short-sc25 Protects Cells from Doxorubicin-Induced Apoptosis

**Figure 6:**
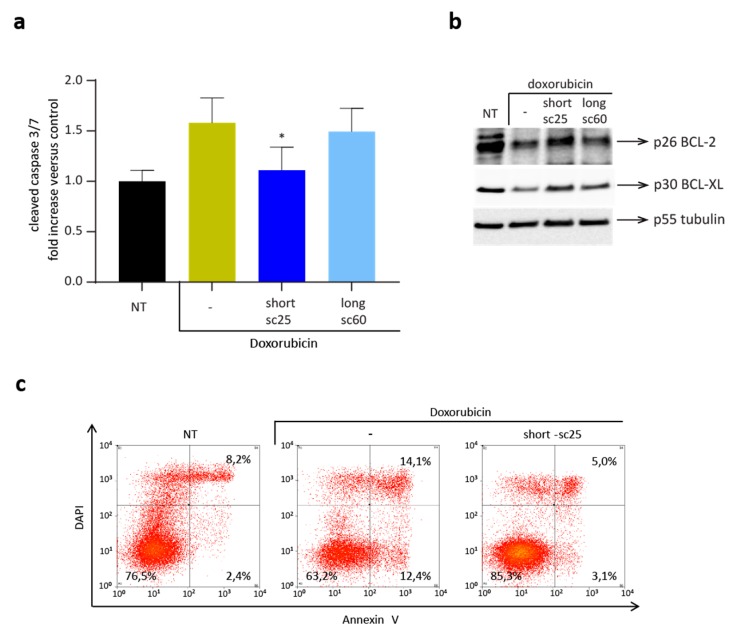
Short-sc25 protects normal cells from apoptosis induced by doxorubicin. (**a**) Analysis of caspase-3 activation in H9C2 cells treated with short-sc25 or long-sc60 (100 nM) for 3 h and then exposed to doxorubicin (25 µM) for 1 h. Graph represents cleaved caspase-3 activity expressed as fold increase versus control. Samples are in triplicate; bars represent standard deviations. * *p* < 0.05 T-test of doxorubicin + short-sc25 treated cells versus doxorubicin alone. NT, Non-Treated cells. (**b**) Immunoblotting analysis of lysates from H9C2 cells treated as in (**a**); activation of anti-apoptotic pathways was determined evaluating BCL-2 and BCL-xL protein expression. Tubulin (p55) was analyzed as loading control. (**c**) Flow cytometry analysis of HPDE cells treated with short-sc25 (100 nM) for 24 h, exposed to doxorubicin (4 µM) for 24 h and then stained with Annexin V-APC and DAPI. Data reported in the figure are representative of at least three experiments.

We then explored the activity of short-sc25, which displays HGF-mimetic properties, within in vitro models of chemotherapy-induced apoptosis, such as H9C2 rat cardiomyoblasts, and human pancreatic duct epithelial (HPDE) cells [21,22]. In H9C2 cells, pre-treatment with short-sc25 significantly hindered the toxic activity of doxorubicin, while long-sc60 was ineffective (Figure 6a). Block of doxorubicin-mediated apoptotic response was confirmed at the molecular level by upregulation of the anti-apoptotic molecules BCL-xL and BCL-2 (Figure 6b), and down-modulation of pro-apoptotic pathways, as assessed by a decrease in the ratio between cleaved and total caspase-3 (Figure S4). In the second model, HPDE cells treated with therapeutic doses of doxorubicin in the presence of short-sc25 showed significant reduction of apoptosis as measured by Annexin V assay (Figure 6c). To standardize the response to short-sc25, we analyzed protection from doxorubicin-induced apoptosis in A549 cells, previously used to evaluate MET phosphorylation and cell motility. In this model, short-sc25 alone showed a ligand-mimetic activity, that was slightly enhanced in combination with HGF (Figure S5).

## 3. Discussion

**Figure 7:**
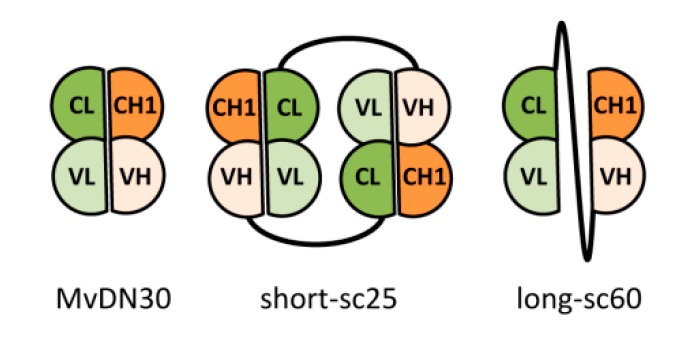
Schematic representation of the hypothesized structure of DN30-derived molecules. VL: variable light domain derived from DN30; CL: constant light domain derived from human IgK light chain; VH: variable heavy domain derived from DN30; CH1: first constant heavy domain derived from human IgG1 heavy chain. Black line represents the amino acid linker.

The MET tyrosine kinase receptor stands upstream of a cascade of events leading to cell scattering, control of proliferation, and protection from apoptosis [1]. Previous work has shown that these responses can be dissociated [23]. This paper describes two engineered antibody fragments derived from the same parental antibody, sharing common properties (e.g., binding and shedding of MET), but endowed with different biological activities (e.g., cell scattering and apoptosis). We designed two single-chain derivatives of the Fab fragment using flexible linkers composed by a diverse array and number of amino acid residues. The electrophoretic analysis of the two derivatives revealed that the molecule with the longer linker (long-sc60) behaves as a monomeric protein, while the one with the shorter linker (short-sc25), under non-reducing conditions, is composed by a mixed population of dimeric and monomeric forms. This result suggests that the presence of the short linker forces the interaction between light and heavy immunoglobulin domains in trans, while the longer linker confers a higher degree of freedom, allowing the spatial rearrangement required for coupling the domains in cis (Figure 7).

When cells are treated with single-chain antibodies for a long time, they induce receptor shedding; as a consequence, surface MET is reduced and HGF-induced activation of MET, as well as of downstream transducers, is impaired. On the contrary, when cells are stimulated with short-sc25 or long-sc60 for a short time, the behavior of the two molecules is different: long-sc60 does not induce any effect, while short-sc25 stimulates MET activation. This is presumably due to the presence of short-sc25 dimers that may concomitantly bind two MET receptors. This interaction results in receptor complex stabilization and kinase activation, ensuing biological responses such as scatter activity and apoptosis protection. Nevertheless, the intensity of receptor phosphorylation obtained upon short-sc25 stimulation is poorer compared to HGF, and thus the downstream signal is impaired; in particular, AKT is less phosphorylated and ERK activation is not sustained. Consequently, short-sc25 agonistic activity mimics HGF in both cell scattering and protection from apoptosis, without reaching its potency.

When cells are stimulated with HGF in the presence of short-sc25, AKT is less phosphorylated compared to HGF alone, while ERK activation is similar. Analyzing the effect of the combination in biological responses, short-sc25 quenches HGF-induced cell motility while it slightly potentiates protection from doxorubicin-induced apoptosis. This is in line with the fact that cell motility is mainly linked to the AKT pathway [24] while protection from apoptosis results from a cooperation between the two transducers [25]. The reason why HGF can fully trigger ERK but not AKT in the presence of short-sc25 could be related to the different kinetic of activation observed for the two transducers upon treatment with the ligand or the antibody. 

In recent years, the therapeutic application of recombinant antibody fragments has gained more and more interest as an alternative option to full-size mAbs. Single-chain derivatives (i.e., small-sized fragments encoded by a single cDNA), are particularly suitable for use as subunits of recombinant fusion proteins directed against multiple molecular targets and/or endowed with different functions [26]. The possibility of expressing a fusion protein containing an antibody moiety as a single polypeptide chain is advantageous for a number of reasons. First, it circumvents the problems related to unbalanced production and/or incorrect combination of antibody heavy and light chains, as well as of other protein subunits included in the fusion. Second, it increases the molecular weight of the antibody fragment, thus reducing the rate of renal clearance. Third, it fixes the stoichiometry of different components in a fusion protein. Finally, it positively affects the production of the recombinant molecule in terms of complexity and/or yield of the entire procedure [27,28].

In the vast majority of cases, cancer-targeted therapy primarily results in blocking cell proliferation, without—or only partially—triggering cell death. Here we demonstrate that MET targeting with the long-sc60 results in activation of the apoptotic program in MET-addicted tumor cells. This could represent a way to improve the therapeutic efficacy. In fact, tumor growth can be controlled by shifting the balance between cell proliferation and cell death in favor of death [29]. Most importantly, only drugs capable of killing cancer cells can achieve a long-lasting therapeutic effect, hopefully leading to eradication of the disease. The anti-apoptotic effect of the long-sc60 could be enhanced by use in combination with inhibitors of anti-apoptotic pathways [30], or with pro-apoptotic drugs such as standard chemotherapy.

A major limitation of chemotherapeutic regimens is the onset of collateral effects inducing damages to a plethora of organs and tissues [31]. It is well known that wild type MET signaling has a protective role in lung fibrosis [32], liver cirrhosis [33], acute pancreatitis [34], and acute kidney injury [35]. Moreover, MET activation protects cardiomyocytes from apoptosis through inhibition of the mitochondrial pathway [12,36,37]. All these evidences suggest the use of MET agonists, such as short-sc25, in association with chemotherapy for the treatment of tumors that are not dependent on MET activation.

## 4. Materials and Methods

### 4.1. Design and Production of Single-Chain Fabs

Single-chain Fabs were designed starting from the sequence of the chimeric Fab MvDN30. The constant domain of the light chain was connected via an amino acid sequence to the variable domain of the heavy chain. Linker sequences are: 5 × GGGGS in short-sc25; GGSSGSGSGSTGTSSS GTGTSAGTTGTSASTSGSGSGGGGGSGGGGSAGGTATAGASSGS in long-sc60. At the C-terminus of each molecule, a triple Strep-tag (GAAWSHPQPEK) and a poly-histidine tag (HHHHHH) were added for protein purification and detection. cDNA synthesis, protein expression, and purification were performed by U-Protein Express BV (Utrecht, The Netherlands). The purified molecules were resolved by polyacrylamide gel electrophoresis (SDS-PAGE) under reducing and non-reducing conditions and stained with the GelCode Blue Stain reagent (Pierce, Waltham, MA, USA). Quantification of the SDS-PAGE bands was performed using ImageJ software.

### 4.2. Cell Culture

A549 human lung adenocarcinoma cells, human pancreatic adenocarcinoma (HPAF-II) cells, SNU-5 human gastric carcinoma cells, human pancreatic duct epithelial (HPDE) cells, and H9C2(2-1) rat myoblast cells were obtained from ATCC/LGC Standards S.r.l. (Sesto San Giovanni, Italy). EBC-1 human lung squamous cell carcinoma lines were purchased from the Japan Cancer Resources Bank. All cell lines were maintained in the culturing conditions suggested by the supplier.

### 4.3. ELISA Binding Assays

The extracellular region of MET fused to the Fc portion of a human IgG (MET-Fc chimera, R&D Systems, Minneapolis, MN, USA) was immobilized on ELISA plates, and increasing concentrations (0–20 nM) of short-sc25, long-sc60, or MvDN30 were added in liquid phase. Binding was revealed using horseradish peroxidase (HRP)-conjugated anti-human K light chain antibodies (Sigma-Aldrich, St. Louis, MO, USA). Colorimetric assay was quantified by the multi-label plate reader VICTOR-X4 (Perkin Elmer Instruments INC., Waltham, MA, USA).

### 4.4. MET Shedding Analysis

Sub-confluent A549 cells were incubated in serum-free medium with increasing concentrations (0.25–1 μM) of single-chain Fabs or MvDN30. After 48 h, the conditioned medium was collected, and cells were lysed with Laemmli buffer. MET protein levels were determined in 25 μg of total cell lysates and in 30 μL of cell culture supernatants by Western blotting using the anti-MET antibody D1C2 to recognize the C-terminal tail of the receptor (Cell Signaling Technology, Danvers, MA, USA) and the DL21 antibody to recognize the MET extracellular domain [16], respectively.

### 4.5. MET Activation Analysis

For evaluation of the antagonist activity of the molecules, sub-confluent A549 cells were incubated with 1 μM single-chain Fabs, MvDN30, or DN30 mAb in serum-free medium. After 24 h, cells were stimulated with 100 ng/mL HGF for 15 min at 37 °C, lysed, and analyzed by Western blot. For evaluation of the agonistic activity, sub-confluent A549 cells were serum-starved for 24 h and then stimulated for 15 min at 37 °C with single-chain Fabs or MvDN30 (0.5–1 μM), DN30 mAb (0.5 μM), or HGF (100 ng/mL) (R&D Systems). For time course experiments, sub-confluent A549 cells were serum-starved for 24 h and then stimulated for 5, 15, or 30 min at 37 °C with short-sc25 (0.5 μM), DN30 mAb (0.5 μM), or HGF (100 ng/mL) (R&D Systems). Total cell lysates were resolved by electrophoresis and analyzed by Western blot.

### 4.6. Western Blot Analysis

Cellular proteins were extracted using Laemmli buffer and protein concentration was measured using Pierce BCA Protein Assay Kit (Thermo Fisher Scientific, Waltham, MA, USA). Cell lysates were resolved by SDS-PAGE and transferred to 0.2 µm nitrocellulose Trans-Blot Turbo TM membranes (Thermo Fisher Scientific). For protein detection, the following antibodies were used according to the protocols supplied by the manufacturers: anti-MET phospho-Tyr1234/1235 (D26, Cell Signaling Technology); anti-MET (D1C2, Cell Signaling Technology); anti-AKT (Cell Signaling Technology); anti-AKT phospho Ser473 (Cell Signaling Technology); anti-ERK1/2 (Cell Signaling Technology); ERK1/2 phospho Thr202/Tyr204 (Cell Signaling Technology); anti-vinculin (clone hVIN-1, Sigma-Aldrich); anti-BIM (C34C5, Cell Signaling Technology); anti-BCL-xL (54H6, Cell Signaling Technology); anti-BCL2 (M19, Santa Cruz Biotechnology Inc., Dallas, TX, USA); anti-alpha actin (Sigma-Aldrich); anti-caspase-3 antibody that detects full length and cleaved caspase-3 forms (Cell Signaling Technology); anti-tubulin (Sigma-Aldrich). HRP-conjugated secondary antibodies were from Jackson Immuno Research Europe (Cambridge, UK). Protein detection and quantification were performed using the ChemiDoc Touch Imaging System (Bio-Rad, Hercules, CA, USA) and the Image Lab software (Bio-Rad).

### 4.7. Growth Assays

SNU-5 and EBC-1 cancer cells were seeded in 96-well plates (2000 cells/well) in the appropriate culture medium in the presence of 10% fetal bovine serum (FBS). After 24 h, cells were treated with increasing concentrations (0–5 μM) of long-sc60, short-sc25, or MvDN30 in fresh culture medium supplemented with 5% FBS. After 72 h of treatment, cell number was determined using CellTiter-Glo Luminescent Cell Assay (Promega, Madison, WI, USA) with a VICTOR X4 multilabel plate reader (Perkin Elmer Inc.).

### 4.8. Scatter Assays

For end-point analysis, HPAF-II cells (8000 cells/well) were seeded in 96-well plates in complete culture medium. After 24 h, cells were incubated in the presence of short-sc25, long-sc60, DN30 mAb, MvDN30 (100 nM), or HGF (5 ng/mL) for 20 h. Cells were fixed with 11% glutaraldehyde and stained with 0.1% Crystal Violet (Sigma-Aldrich). For real-time cell motility assay, HPAF-II cells (8000 cells/well) were seeded in E-plates (Roche Diagnostics, Mannheim, Germany) in complete culture medium and treated as above. Electrical impedance was monitored continuously for 24 h using an X-Celligence RTCA device (Roche Diagnostic). The electronic readout of cell-sensor impedance is displayed in real-time as cell index, a value directly influenced by cell shape and spreading. The addition of HGF induced cell flattening and dissociation that resulted in an increase of the cell index.

### 4.9. Analysis of Caspase 3 Enzymatic Activity

EBC-1 cells were seeded in 96-well plates (2000 cells/well) in the appropriate culture medium in the presence of 10% FBS. After 24 h, cells were treated with increasing concentrations (0–10 μM) of long-sc60 or short-sc25 in fresh culture medium supplemented with 5% FBS. After 72 h caspase enzymatic activity was evaluated using Caspase-Glo^®^ 3/7 assay system (Promega). H9C2 cardiomyocytes were seeded in 96-well plates (5000 cell/well) in the appropriate culture medium in the presence of 10% FBS. After 24 h, the medium was replaced with serum-free medium. The day after, cells were treated with short-sc25 or long-sc60 (100 nM). After 3 h, doxorubicin (25 µM) was added, and 1 h later the medium was replaced with fresh medium for 2 h. Caspase activation was detected as described above.

### 4.10. Analysis of Apoptosis Markers by Immunoblotting

EBC-1 cells were seeded in 6-well plates (300,000 cells/well) in the appropriate cell culture medium. After 24 h of treatment with short-sc25 or long-sc60 (0.5 µM) in complete medium cell proteins were extracted with Laemmli buffer and analyzed by Western blot. H9C2 cells were plated in 10 cm diameter dishes (450,000 cells/dish) in complete cell culture medium. After 24 h, the medium was replaced with 0.5% FBS medium for another day. The cells were treated with short-sc25 or long-sc60 (100 nM) for 3 h and then exposed to doxorubicin (25 µM) for 1 h. After 24 h recovery in 0.5% FBS medium, cell proteins were extracted with Laemmli buffer and analyzed by Western blot.

### 4.11. Analysis of Annexin V-Positive Cells

SNU-5 and EBC-1 cancer cells (300,000 cells/well) were seeded in 6-well plates in complete culture medium. The following day, cells were treated with long-sc60 (0.5 µM). After 48 h, cells were washed with PBS, stained with Annexin V-APC (Thermo Fisher Scientific) and DAPI (Roche Diagnostic). Flow cytometry analysis was performed using a CyAN ADP apparatus (Dako, Santa Clara, CA, USA), and data were elaborated with Summit 4.3 software (Dako). HPDE cells (500,000 cells/well) were seeded in 6-well plates in complete cell culture medium. The following day, cells were treated with short-sc25 (100 nM). After 24 h cells were exposed to doxorubicin (4 µM) for further 24 h. Cells were then stained with Annexin V-APC and DAPI and analyzed as described above.

### 4.12. Statistical Analysis

Average and standard deviation (SD) were calculated using Microsoft Office Excel 2010 software (Microsoft Corporation, Redmond, WA, USA). To calculate Kd values, data from ELISA assays were analyzed and fitted according to nonlinear regression and one site binding hyperbola curve, using GraphPad Prism software (GraphPad Software, San Diego, CA, USA). To calculate inhibitory concentration 50 (IC_50_) values, data from growth assays were analyzed and fitted according to nonlinear regression, dose-response inhibition curve, using GraphPad Prism software. Statistical significance was determined using the two-tailed Student’s t-test. A value of *p* ≤ 0.05 was considered significant.

## 5. Conclusions

In this study, we demonstrated the possibility of achieving selective modulation of the apoptotic response by generating specific MET-targeting antibody fragments. Tailoring the molecular engineering strategy, we generated antibodies with opposite biological activity, potentially functional either to control tumor growth or to protect healthy tissue from drug toxicity. Both applications could hopefully contribute to improve the clinical outcome and the quality of life of cancer patients.

## Supplementary Materials

The following are available online at https://www.mdpi.com/2072-6694/12/3/741/s1, Figure S1: Short-sc25 induces MET activation, Figure S2: Activity of short-sc25 in combination with HGF, Figure S3: Short-sc25 induces scattering of A549 cells, Figure S4: Short-sc25 inhibits caspase-3 activation in H9C2 cells treated with doxorubicin, Figure S5: Short-sc25 protects A549 cells from doxorubicin-induced apoptosis.
