# Supplementary material for: Molecular Engineering Strategies Tailoring the Apoptotic Response to a MET Therapeutic Antibody
Source: Cancers (Basel). 2020 Mar 21;12(3):741. doi: 10.3390/cancers12030741 (PMC7140090; doi:10.3390/cancers12030741)
Supplement: Supplementary file 1 [file cancers-12-00741-s001.zip › cancers-742035 supplementary final/cancers-742035 Weatern Blot.pptx]

## Slide 1
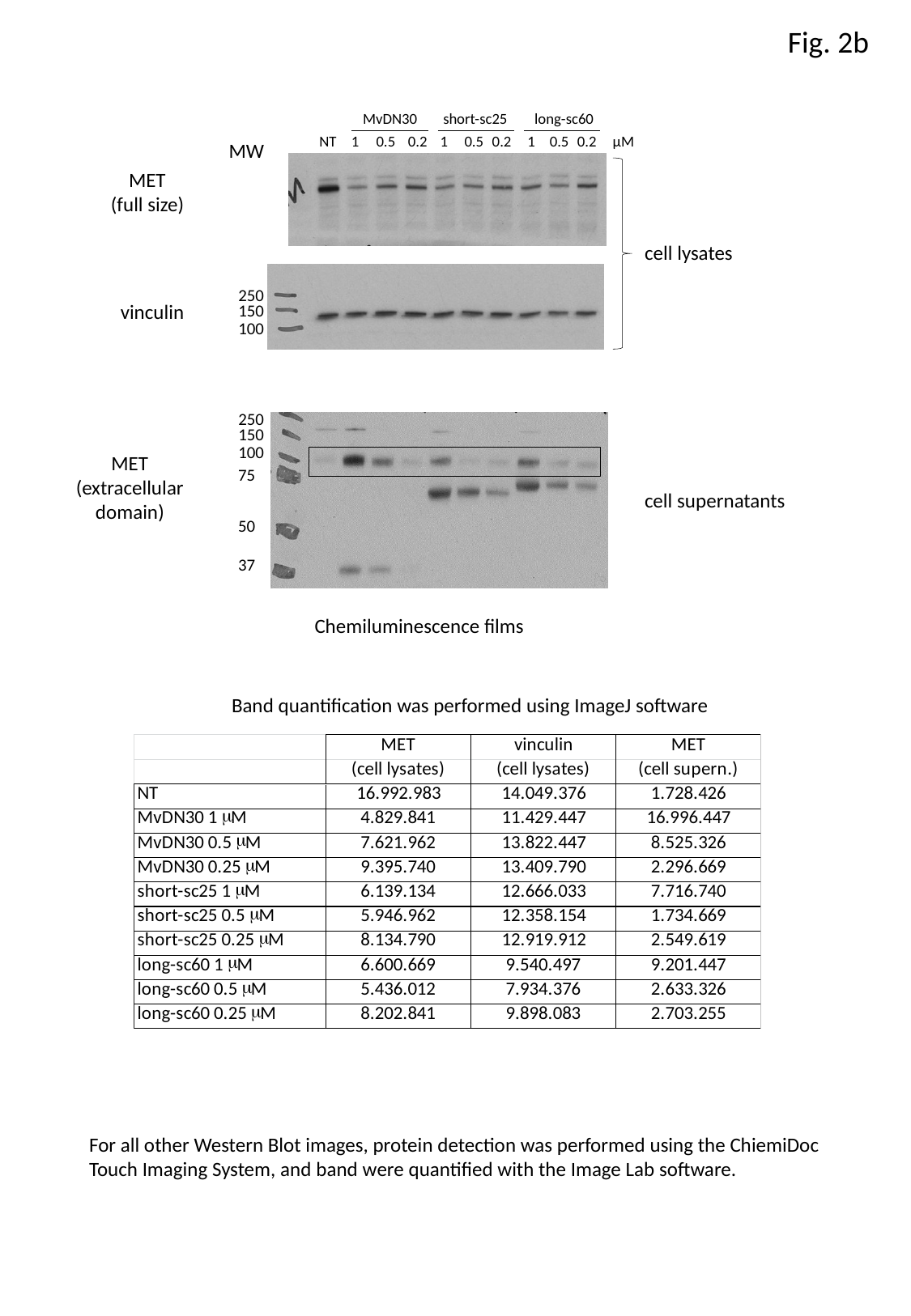

Fig. 2b
MvDN30
short-sc25
long-sc60
NT
1
0.5
0.2
1
0.5
0.2
1
0.5
0.2
μM
MET
(full size)
cell lysates
vinculin
MET
(extracellular
domain)
cell supernatants
Chemiluminescence films
MW
250
150
100
250
150
100
75
50
37
Band quantification was performed using ImageJ software
For all other Western Blot images, protein detection was performed using the ChiemiDoc Touch Imaging System, and band were quantified with the Image Lab software.

## Slide 2
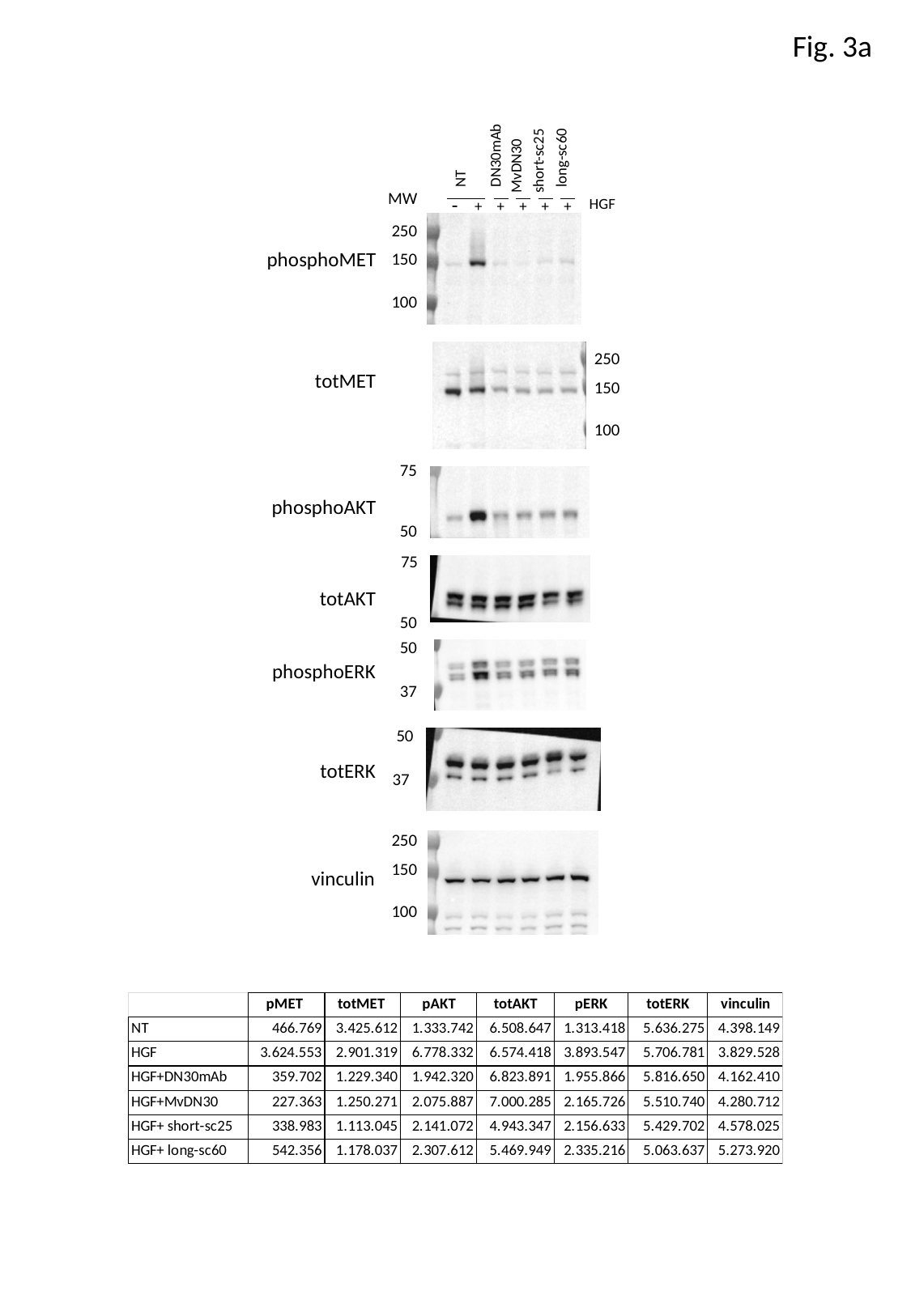

Fig. 3a
short-sc25
DN30mAb
long-sc60
MvDN30
NT
-
HGF
+
+
+
+
+
phosphoMET
totMET
phosphoAKT
totAKT
phosphoERK
totERK
vinculin
MW
250
150
100
250
150
100
75
50
75
50
50
37
50
37
250
150
100

## Slide 3
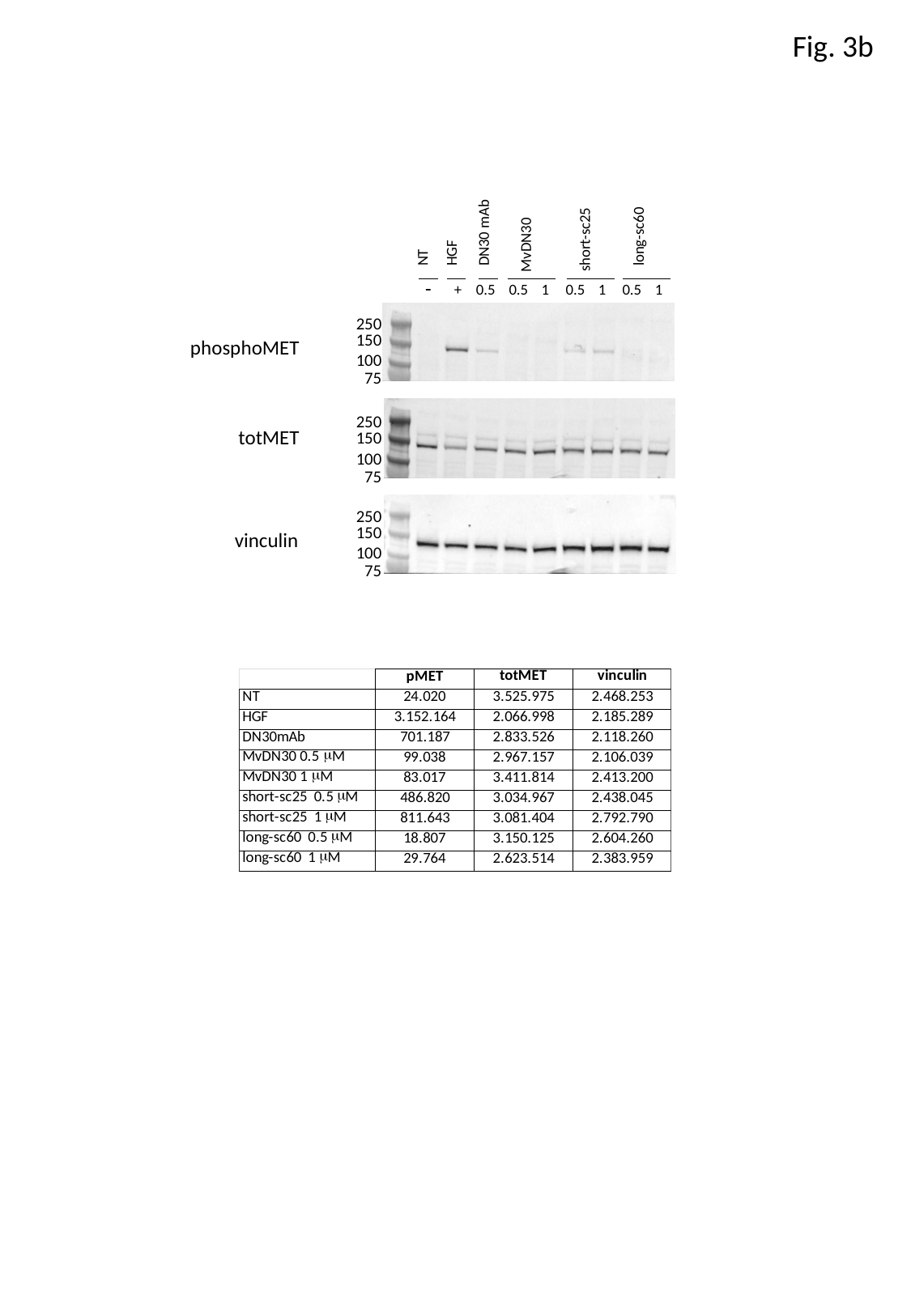

Fig. 3b
DN30 mAb
short-sc25
long-sc60
MvDN30
HGF
NT
-
+
0.5
0.5
1
0.5
1
0.5
1
phosphoMET
totMET
vinculin
250
150
100
75
250
150
100
75
250
150
100
75

## Slide 4
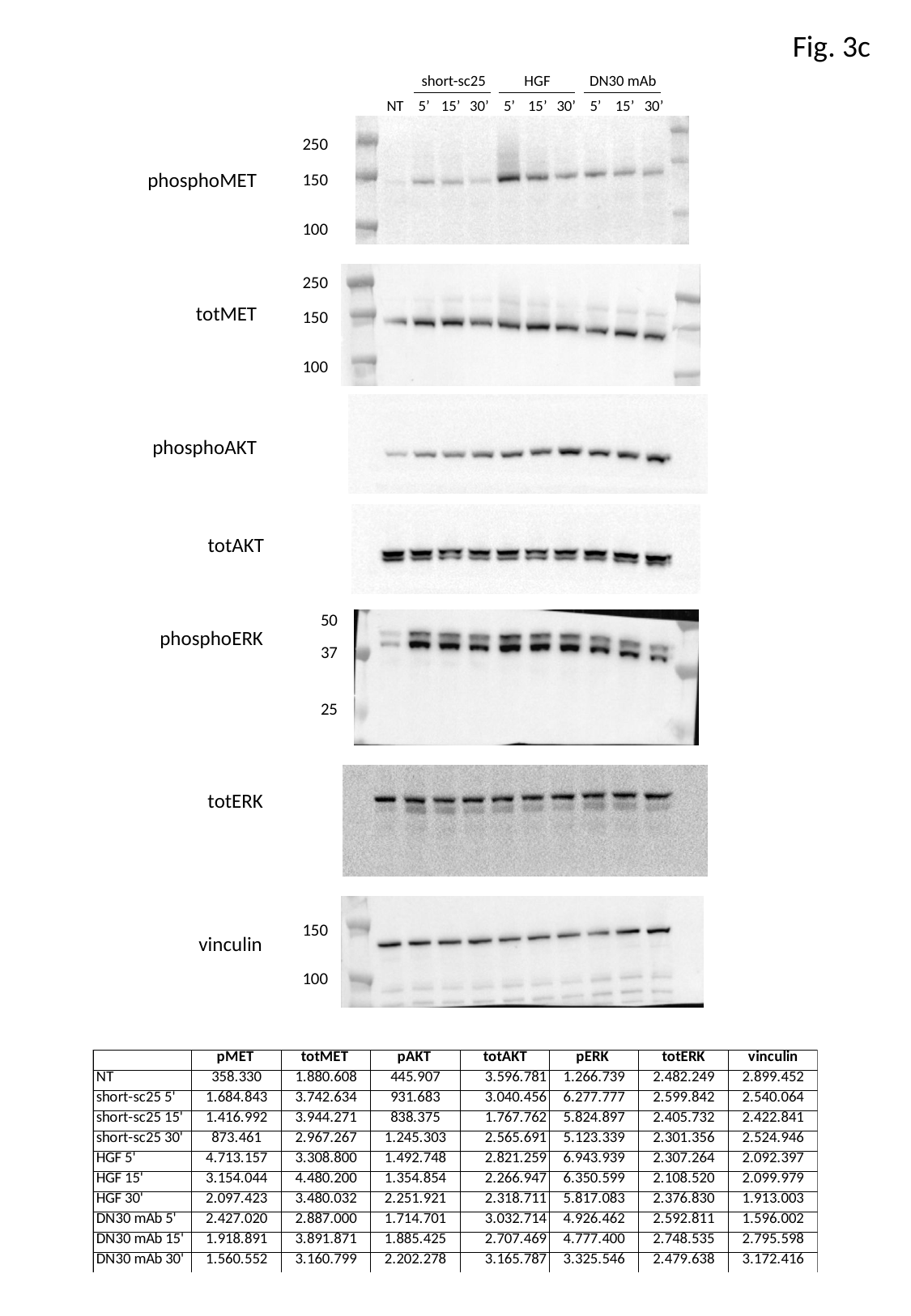

Fig. 3c
short-sc25
HGF
DN30 mAb
NT
5’
15’
30’
5’
15’
30’
5’
15’
30’
phosphoMET
totMET
phosphoAKT
totAKT
phosphoERK
totERK
vinculin
250
150
100
250
150
100
50
37
25
150
100

## Slide 5
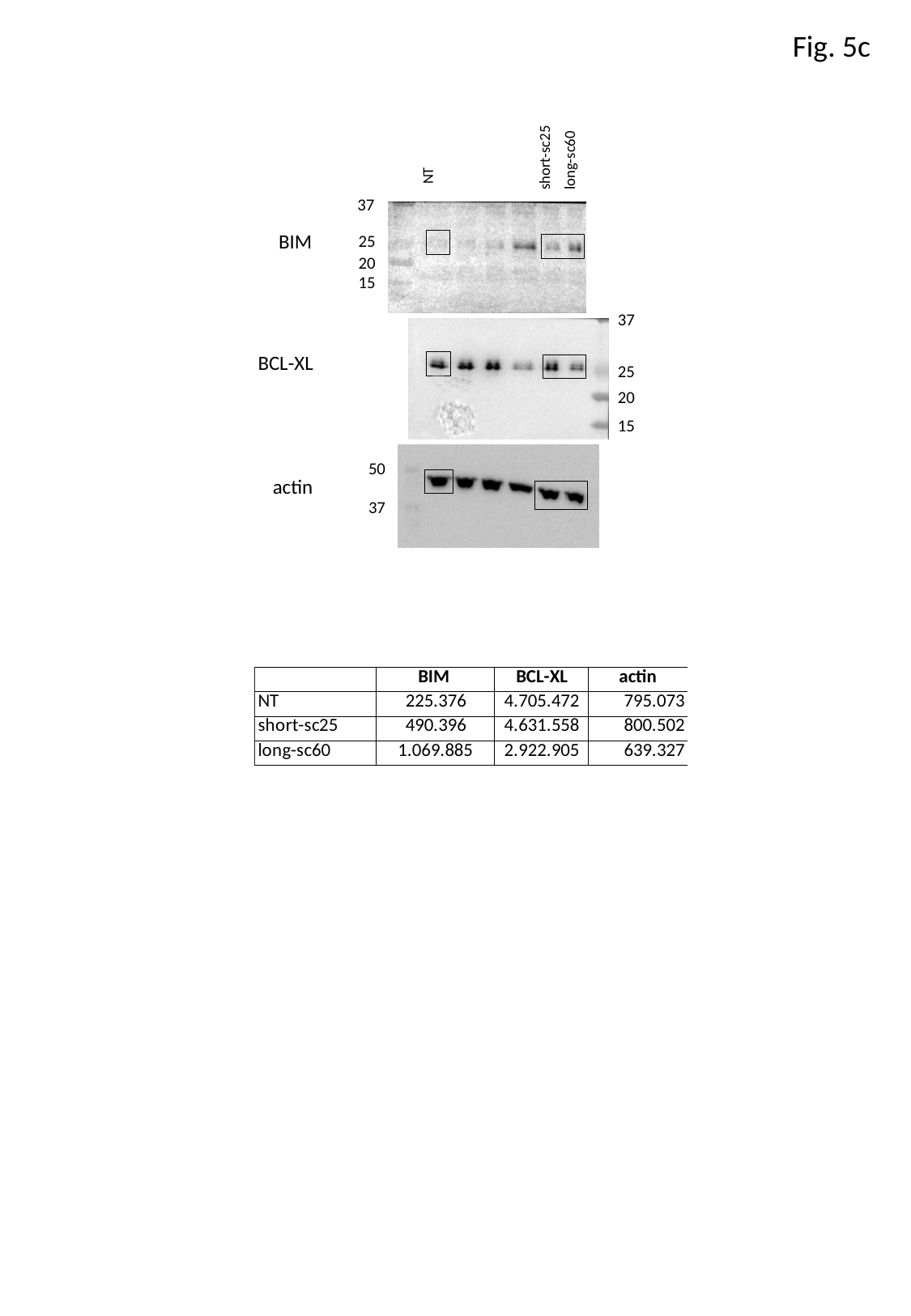

Fig. 5c
short-sc25
long-sc60
NT
BIM
BCL-XL
actin
37
25
20
15
37
25
20
15
50
37

## Slide 6
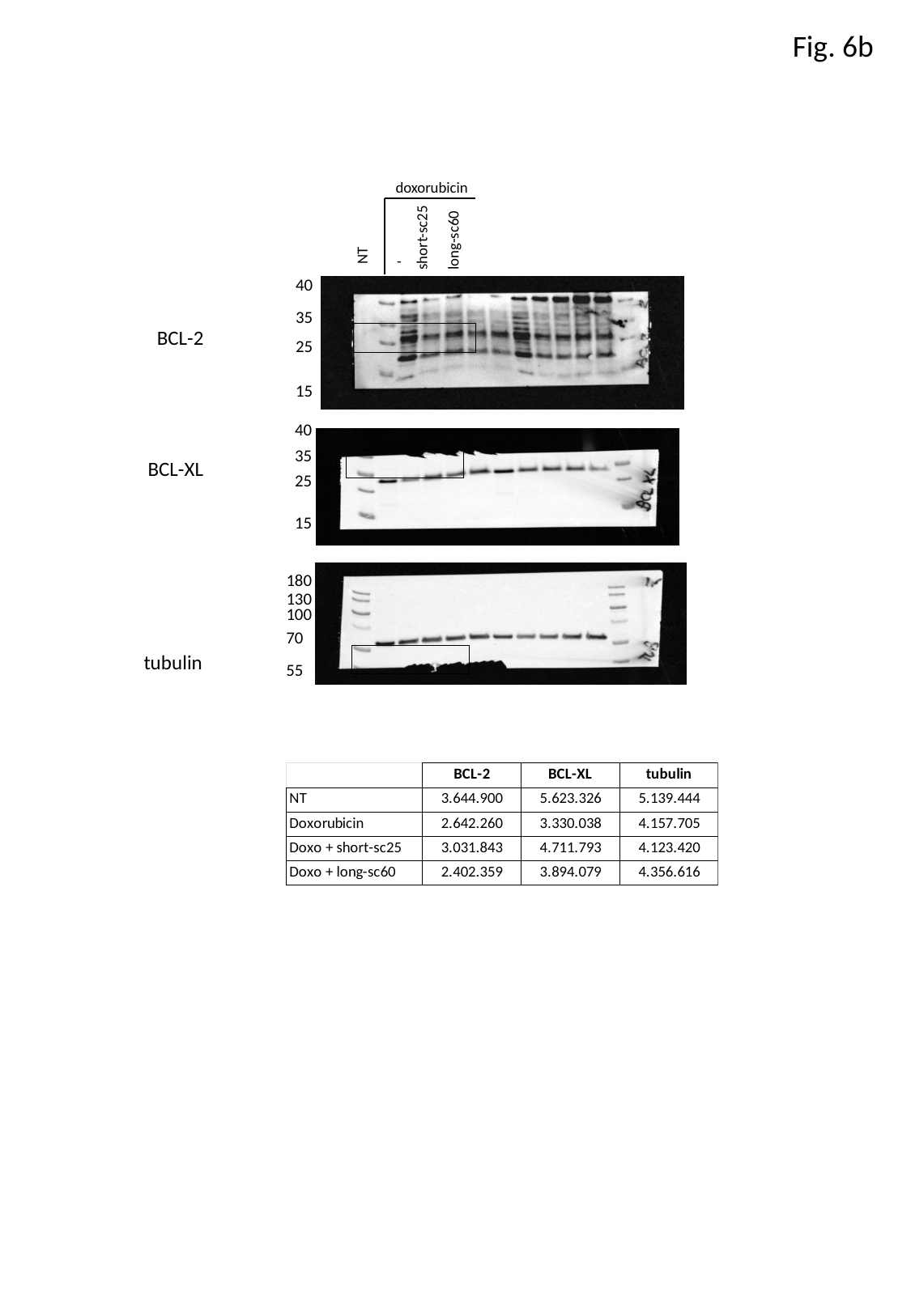

Fig. 6b
doxorubicin
short-sc25
long-sc60
-
NT
40
35
25
15
BCL-2
40
35
25
15
BCL-XL
180
130
100
70
tubulin
55

## Slide 7
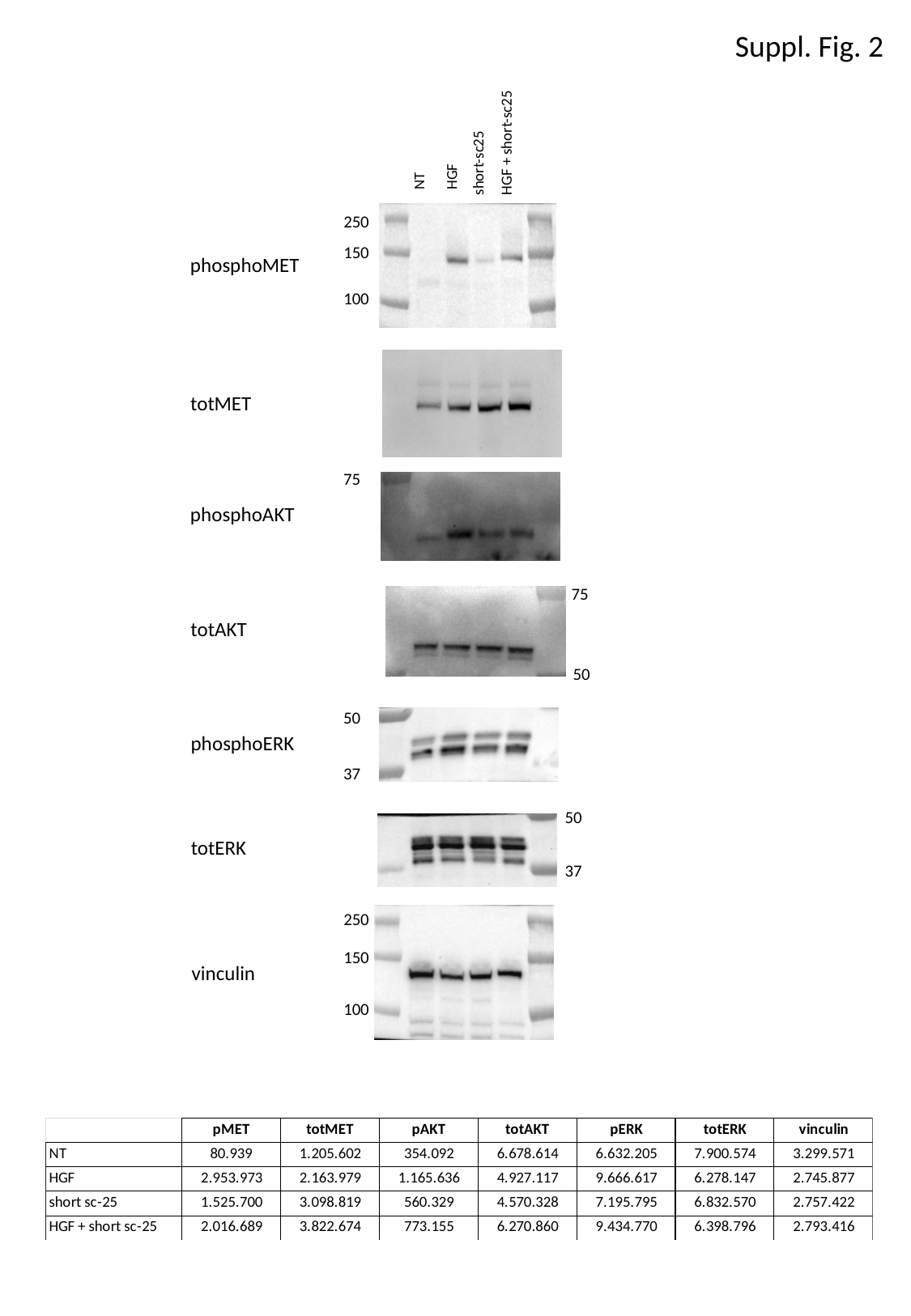

Suppl. Fig. 2
HGF + short-sc25
short-sc25
HGF
NT
250
150
phosphoMET
100
totMET
75
phosphoAKT
75
totAKT
50
50
phosphoERK
37
50
totERK
37
250
150
vinculin
100

## Slide 8
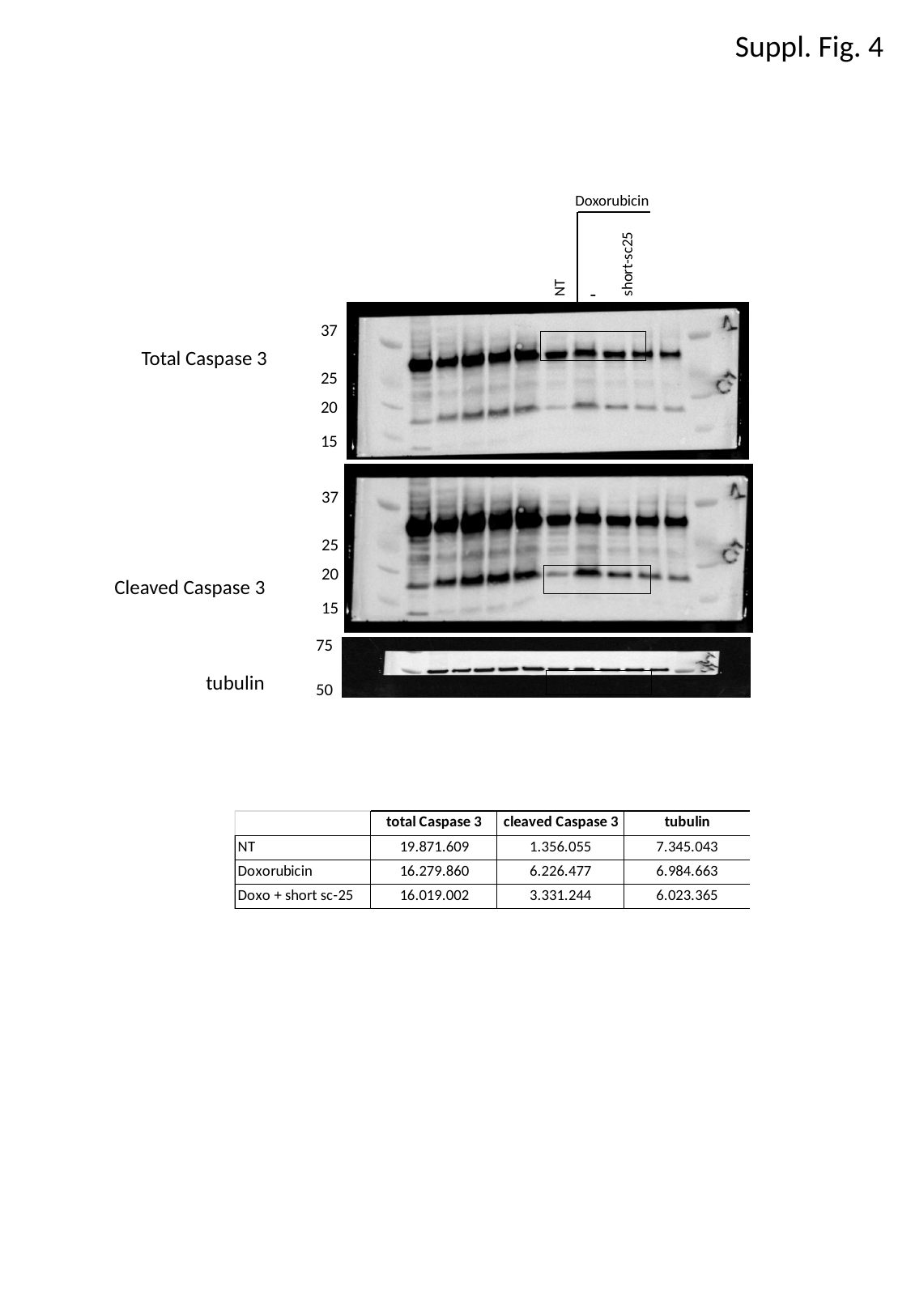

Suppl. Fig. 4
Doxorubicin
short-sc25
NT
-
Total Caspase 3
Cleaved Caspase 3
tubulin
37
25
20
15
37
25
20
15
75
50
