# Supplementary material for: Molecular Engineering Strategies Tailoring the Apoptotic Response to a MET Therapeutic Antibody
Source: Cancers (Basel). 2020 Mar 21;12(3):741. doi: 10.3390/cancers12030741 (PMC7140090; doi:10.3390/cancers12030741)
Supplement: Supplementary file 1 [file cancers-12-00741-s001.zip › cancers-742035 supplementary final/cancers-742035-supplementary final.pdf]

Supplementary materials:

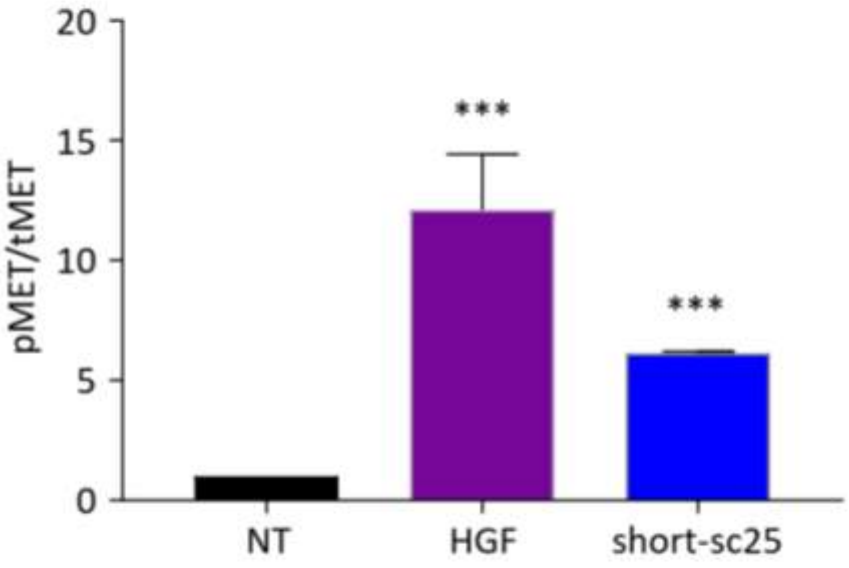

Figure S1. Short-sc25 induces MET activation. Sub-confluent A549 cells were serum-starved for 24 h and then stimulated for 15 min at 37 °C with HGF (100 ng/mL) or short-sc25 (0.5 µM). Total cell lysates were resolved by electrophoresis and analyzed by Western blot with anti-MET phospho-Tyr1234/1235 or anti-MET antibodies. Graph represents phospho-MET versus total MET ratio; values were determined by densitometry analysis of immunoblot signals obtained from 3 independent experiments. *** $p < 0.001$ T-test of Non-Treated (NT) cells versus HGF-treated or short-sc25 treated cells.

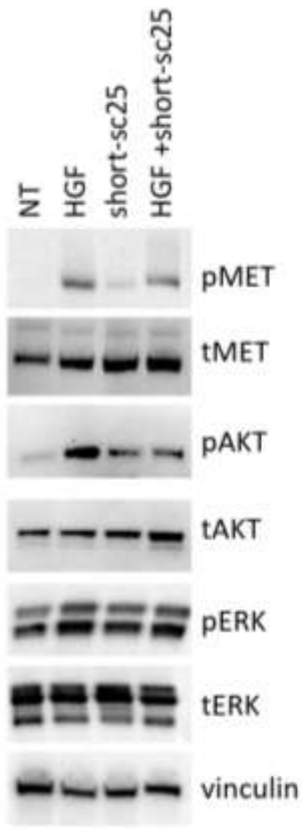

Figure S2. Activity of short-sc25 in combination with HGF. Serum-starved A549 cells were stimulated for 15 min with HGF (100 ng/mL), short-sc25 (0.5 µM), or both molecules in combination. Total cell lysates were analyzed by immunoblotting. Vinculin was used as loading control. MET phosphorylation and total MET levels were measured using anti-MET antibodies recognizing the major phosphorylation site (Tyr1234/1235) or the C-terminal tail of the molecule, respectively. AKT phosphorylation was measured using anti-AKT antibodies recognizing the phosphorylated Ser473, and ERK phosphorylation was measured using anti-ERK antibodies recognizing the phosphorylated Thr202/Tyr204.

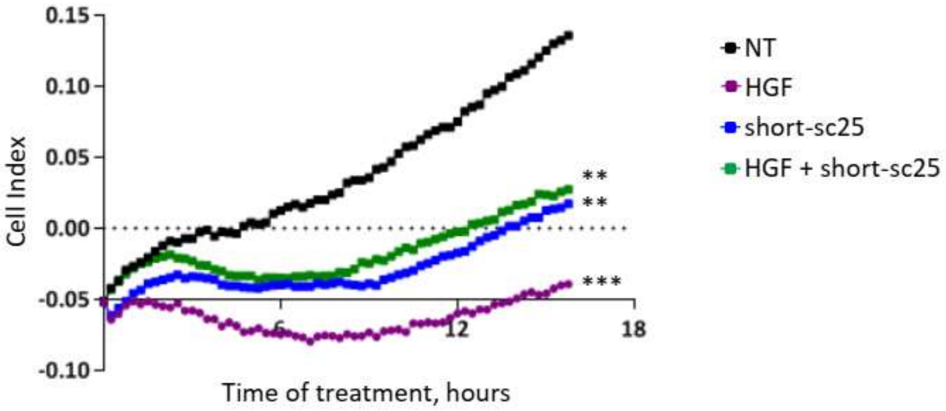

Figure S3. Short-sc25 induces scattering of A549 cells. A549 cells were seeded in E-plates (Roche Diagnostics, Mannheim, Germany, 8,000 cells/well) in complete culture medium, and treated with HGF (5 ng/mL), short-sc25 (100nM), or the combination of the two molecules. Electrical impedance was monitored continuously for 16 hrs using an X-Celligence RTCA device (Roche Diagnostic). The electronic readout of cell–sensor impedance is displayed in real-time as Cell Index, a value directly influenced by cell shape and spreading. Addition of HGF induces cell shrinkage and dissociation [1], that results in a decrease of cell index. NT: Non-Treated cells. Data reported in the plots are representative of at least three independent experiments. ***: $p \leq 0.001$; **: $p \leq 0.01$ T- test of treated versus untreated cells at the end of the experiments..

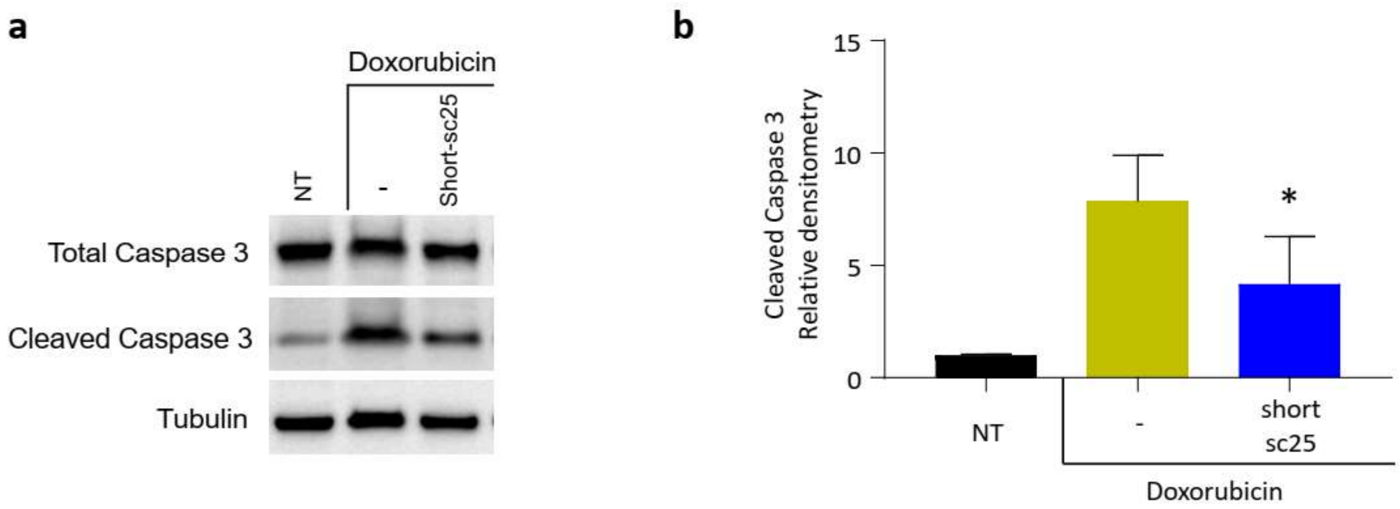

Figure S4. Short-sc25 inhibits caspase-3 activation in H9C2 cells treated with doxorubicin**.** (**a**) Immunoblotting analysis of lysates from H9C2 cells treated with short-sc25 (100 nM) for 3 h and then exposed to doxorubicin (25 μM) for 1hr. Tubulin (p55) was analyzed as loading control. (**b**) Graph representing cleaved versus total caspase-3 ratio, relative to Non-Treated (NT) cells. Values were determined by densitometry analysis of immunoblot signals obtained from 3 independent experiments. * $p < 0.05$ T-test of doxorubicin + short-sc25 treated cells versus doxorubicin alone.

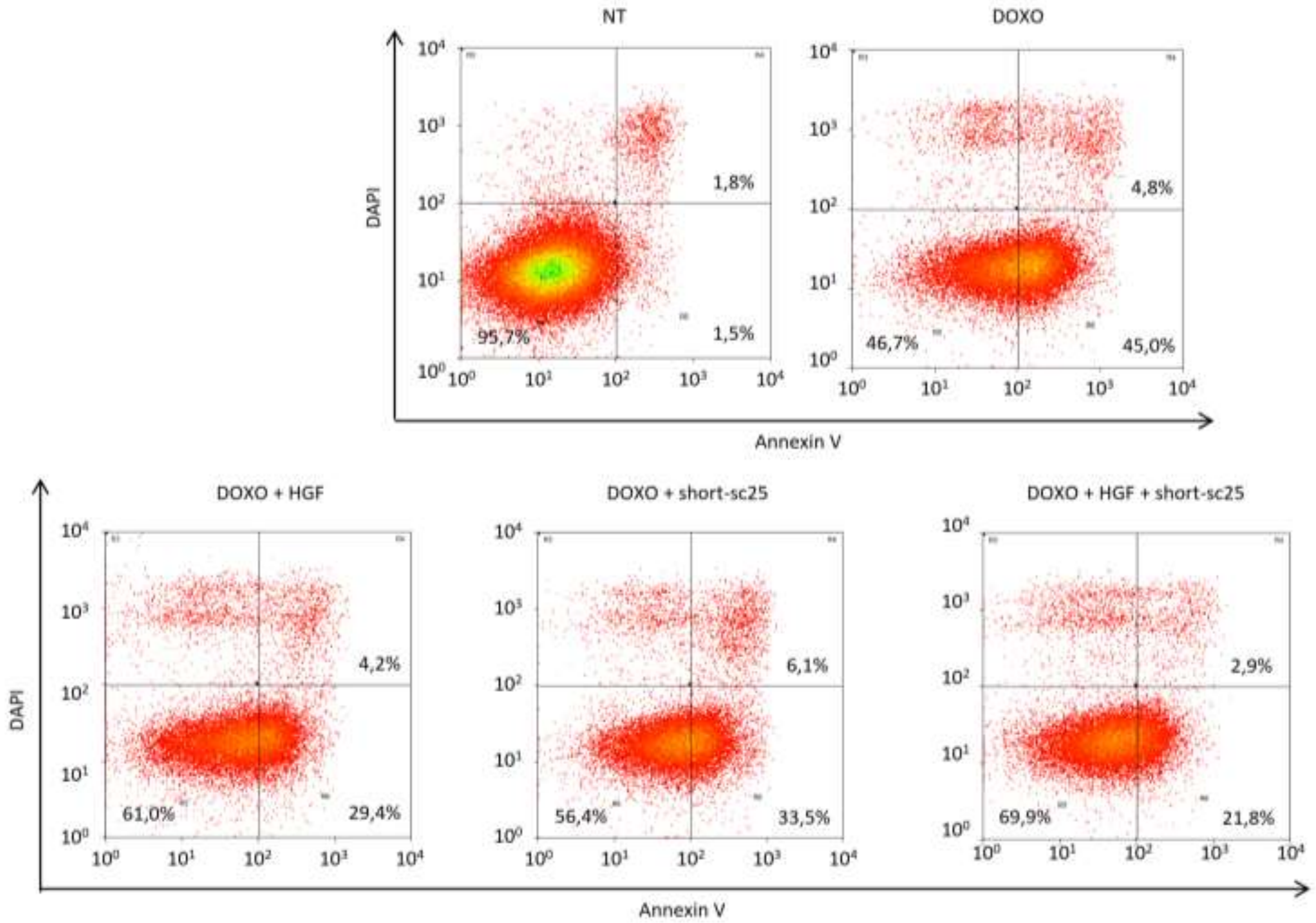

Figure S5. Short-sc25 protects A549 cells from doxorubicin-induced apoptosis. A549 cells were seeded in 6-well plates (300,000 cells/well) in complete cell culture medium. The following day, cells were starved for 24 h and then treated with HGF (50 ng/mL), short-sc25 (0,5 µM) or the combination of the two molecules for further 24 hrs. Cells were then exposed to doxorubicin (4 µM) and after 24 h stained with Annexin V-APC and DAPI. Analysis was performed using a CyANADP apparatus (Dako, Santa Clara, CA), and data were elaborated with Summit 4.3 software (Dako).

References

1 Buus, R.; Faronato, M.; Hammond, D.E.; Urbé, S. and Clague, M.J. Deubiquitinase activities required for hepatocyte growth factor-induced scattering of epithelial cells. *Curr. Biol.* **2009**,*19*, 1463–1466
